# The role of personality and psychopathology in people with migraines

**DOI:** 10.1192/j.eurpsy.2023.1299

**Published:** 2023-07-19

**Authors:** I. Georgiadis, K. Fountas, F. Malli, E. Dragioti, M. Gouva

**Affiliations:** 1 1st Neurosurgical Department, Iaso Thessalias Private Hospital; 2 Department of Neurosurgery, Faculty of Medicine, University of Thessaly; 3 Respiratory Disorders Laboratory, Faculty of Nursing, University of Thessaly, Larissa; 4 Laboratory of Psychology of Patients, Families & Health Professionals, University of Ioannina – School of Health Sciences; 5 Laboratory of Psychology of Patients, Families & Health Professionals, University of Ioannina-School of Health Sciences, Ioannina, Greece

## Abstract

**Introduction:**

Several studies have shown that the relationship between migraine and psychological factors is significant, but few have evaluated the relationship between these psychological factors and patients’ social life.

**Objectives:**

Exploring the role of personality and psychopathology in people with migraines.

**Methods:**

The sample consisted of 180 people, more specifically 140 people from the general population and 40 people who have been diagnosed with migraine and receiving treatment for migraine, who completed the following questionnaires voluntarily and anonymously: a) Migraine Experience Questionnaire and Headache Impact Test-6 (HIT-6), b) Eysenck Personality Questionnaire, c) Symtom Checklist 90-R (SCL-90) and socio-demographic and self-reported questionnaire.

**Results:**

Patients scored higher somatization rates (10.21 ± 8.08), phobic anxiety (3.00 ± 4.45), neuroticism (4.09 ± 1.37), than people from the general population who scored lower somatization rates (14.63 ± 3.12), Phobic anxiety (5.28 ± 1.89), Neuroticism (6.53 ± 2.12), with a statistically significant difference between them (p = 0.001), (p = 0.002), (p = 0.000), respectively.

**Conclusions:**

Patients with symptoms of migraine show statistically higher rates of somatization, phobic anxiety, neuroticism and further study is considered necessary.

**Disclosure of Interest:**

None Declared

